# Aortic Stiffness Hysteresis in Isolated Mouse Aortic Segments Is Intensified by Contractile Stimuli, Attenuated by Age, and Reversed by Elastin Degradation

**DOI:** 10.3389/fphys.2021.723972

**Published:** 2021-09-28

**Authors:** Sofie De Moudt, Arthur Leloup, Paul Fransen

**Affiliations:** Physiopharmacology, Department Pharmaceutical Sciences, University of Antwerp, Antwerpen, Belgium

**Keywords:** aortic stiffness, aging, pressure-dependent hysteresis, viscosity, constriction

## Abstract

**Aim:** Cyclic stretch of vascular tissue at any given pressure reveals greater dimensions during unloading than during loading, which determines the cardiac beat-by-beat hysteresis loop on the pressure-diameter/volume relationship. The present study did not focus on hysteresis during a single stretch cycle but investigated whether aortic stiffness determined during continuous stretch at different pressures also displayed hysteresis phenomena.

**Methods:** Aortic segments from C57Bl6 mice were mounted in the Rodent Oscillatory Set-up for Arterial Compliance (ROTSAC), where they were subjected to high frequency (10 Hz) cyclic stretch at alternating loads equivalent to a constant theoretical pulse pressure of 40 mm Hg. Diastolic and systolic diameter, compliance, and the Peterson elastic modulus (E_p_), as a measure of aortic stiffness, was determined starting at cyclic stretch between alternating loads corresponding to 40 and 80 mm Hg, at each gradual load increase equivalent to 20 mm Hg, up to loads equivalent to pressures of 220 and 260 mm Hg (loading direction) and then repeated in the downward direction (unloading direction). This was performed in baseline conditions and following contraction by α_1_ adrenergic stimulation with phenylephrine or by depolarization with high extracellular K^+^ in aortas of young (5 months), aged (26 months) mice, and in segments treated with elastase.

**Results:** In baseline conditions, diastolic/systolic diameters and compliance for a pulse pressure of 40 mm Hg were larger at any given pressure upon unloading (decreasing pressure) than loading (increasing pressure) of the aortic segments. The pressure-aortic stiffness (E_p_) relationship was similar in the loading and unloading directions, and aortic hysteresis was absent. On the other hand, hysteresis was evident after activation of the VSMCs with the α_1_ adrenergic agonist phenylephrine and with depolarization by high extracellular K^+^, especially after inhibition of basal NO release with L-NAME. Aortic stiffness was significantly smaller in the unloading than in the loading direction. In comparison with young mice, old-mouse aortic segments also displayed contraction-dependent aortic hysteresis, but hysteresis was shifted to a lower pressure range. Elastase-treated segments showed higher stiffness upon unloading over nearly the whole pressure range.

**Conclusions:** Mouse aortic segments display pressure- and contraction-dependent diameter, compliance, and stiffness hysteresis phenomena, which are modulated by age and VSMC-extracellular matrix interactions. This may have implications for aortic biomechanics in pathophysiological conditions and aging.

## Introduction

When the left ventricle of the heart ejects its blood into the circulation, the aorta undergoes large deformations to buffer the volume of the ejected blood. Thereby, the aorta acts as an elastic reservoir to store energy when dilating and to release this energy by elastic recoil during diastole. Evolution designed the aorta to optimize this heart-vessel coupling. The highest relative elastin content in the vascular tree is found in the blood vessels closer to the heart. These elastic arteries are characterized by stress-strain relationships with stress only gradually increasing with elevated pressure in the low pressure range (Armentano et al., 1995; Jesudason et al., 2007; Ratz, 2016). Although the relative VSMC content of the thoracic aorta is lower compared with any other part of the arterial tree, VSMCs are still the predominant cell type in the aortic wall thoracic aorta (Dinardo et al., 2014) and may play a determinant role in biomechanical behavior of the aorta (Qiu et al., 2010; Min et al., 2012; Saphirstein et al., 2013; Leloup et al., 2019). The VSMCs in the aorta are embedded in a plexus of elastin, collagen, and proteoglycans. The orientation and the quantity of these extracellular matrix (ECM) components are responsible for the arterial passive mechanical properties and crucially determine the non-linear stretch-strain relationship of the aorta (Wagenseil and Mecham, 2009, 2012; Charalambous et al., 2017). With aging, the compliance of the aorta slowly decreases, and the pulse-dampening properties are attenuated. It has been described that besides passive adaptations of the vessel wall, involving changes in the extracellular matrix (ECM), also, the intrinsic mechanical properties of the vascular smooth muscle cells (VSMCs) contribute to increased vascular stiffness in aging. In this process, both β_1_-integrin and α-smooth muscle actin are likely major players in the age-dependent stiffening of VSMC (Qiu et al., 2010; Gao et al., 2014).

Elastic arteries such as the aorta are, however, not purely elastic but also exhibit viscous behavior. At any given pressure, they display greater dimensions during unloading than during loading, which determines a hysteresis loop on the pressure/volume relationship, a process known for a long time (Remington, 1955; Bergel, 1961a,b). This means that, on a cardiac beat-by-beat scale, part of the energy stored by the arterial wall during dilation (diastole to systole) is dissipated within the arterial wall during recoil (systole to diastole) (Bauer et al., 1979; Busse et al., 1981; Barra et al., 1993; Armentano et al., 1995; Boutouyrie et al., 1997). The pulsatile flow of the heart transforms to a nearly continuous flow in the peripheral capillaries. An *in vivo* and *in vitro* study of rat abdominal aorta revealed that the arterial wall viscosity was strongly influenced by steady and pulsatile mechanical load, but not by smooth muscle tone (Boutouyrie et al., 1998). Using cellular micro-biaxial stretching microscopy, Win et al. (2018) found that large-strain anisotropic stress caused hysteresis of individual VSMCs. This hysteresis was strongly dependent on load orientation and actin organization. When stretched along the primary fiber alignment, VSMCs exhibited hysteresis with unloading stresses smaller than loading stresses, while stretch in the transverse direction led to reverse hysteresis.

Arterial stiffness is inherently coupled to blood pressure, complicating the interpretation of arterial stiffness measurements in the *in vivo* situation. *In vivo*, acute manipulation (pharmacological or mechanical) of arterial pressure allows to compare arterial stiffness at the same level of pressure and to investigate the pressure sensitivity of arterial stiffness (Butlin et al., 2020). As far as we know, an *ex vivo* stiffness-pressure relationship, comparing isobaric aortic stiffness while increasing and decreasing pressure (as performed in *in vivo* studies), has never been studied before. In the present study, isolated mouse aortic segments were cyclically stretched in a highly controllable extracellular environment and at high frequency (10 Hz) to simulate the physiological heart rate in mice with the Rodent Oscillatory Tension Set-up to study Arterial Compliance (ROTSAC), an in-house-developed setup to study biomechanical properties of *ex vivo* intact aortic rings (Leloup et al., 2016). We did not intend to study classical hysteresis phenomena as observed in the beat-to-beat aortic diameter or lumen cross-sectional area-pressure relationship but aimed to study hysteresis phenomena in aortic compliance or stiffness pressure relationships when slower stepwise pressurization/depressurization was superimposed to the cyclic pulsation of the segments. Thereby, we hypothesized that VSMC contraction, mouse age, and extracellular matrix affected the pressure-stiffness relationship differently in the pressurization vs. depressurization direction in *ex vivo* mouse aortic segments.

## Methods and Materials

### Animals

All animals were bred and housed in the University of Antwerp animal facility in standard cages with 12/12-h light-dark cycles, with free access to regular chow and tap water. This study was approved by the Ethical Committee of the University of Antwerp (ECD2017-11), and all experiments were performed conforming to the Guide for the Care and Use of Laboratory Animals, published by the US National Institutes of Health (NIH Publication No. 85–23, revised 1996). The following C57Bl/6 (original substrain C57Bl/6JRj, Janvier Labs, France) male mouse animal groups were used: group 1, 4 to 7 months of age (*n* = 7, 5.8 ± 1.3 months), group 2, 5 months of age (*n* = 8, further named young, 5. ± 0.1 months), group 3, 26 months of age (*n* = 5, further named old, 26. ± 0.2 months), and group 4, 6 months of age (*n* = 5, further named elastase group, 6. ± 0.1 months). Comparisons between groups of mice only occurred between groups 2 and 3 mice. Both groups contained initially eight mice. Sample size was determined *a priori* with a statistical power of 90%, an effect size of 20% (pairwise comparison), and a coefficient of variation of 20%. The family-wise type I error was controlled at 5%. These figures (spread and effect size) were derived from historical data available at the lab. The study ended with unequal group sizes, because, in the aged group, three mice died before the *ex vivo* experiments started.

### Aortic Tissue Preparation

Mice were euthanized by perforating the diaphragm while under deep anesthesia [sodium pentobarbital (Sanofi, Belgium), 250 mg kg-1, intraperitoneal]. The thoracic aorta was carefully removed and stripped of adherent tissue. Starting a ~2 mm distally from the aortic arch, the descending aorta was cut into four segments of 2-mm length and immersed in Krebs Ringer (KR) solution (37°C, 95% O_2_/5% CO_2_, pH 7.4) containing (in mM): NaCl 118, KCl 4.7, CaCl_2_ 2.5, KH_2_PO_4_ 1.2, MgSO_4_ 1.2, NaHCO_3_ 25, CaEDTA 0.025, and glucose 11.1.

### Rodent Oscillatory Set-Up for Arterial Compliance

**Figure 1:**
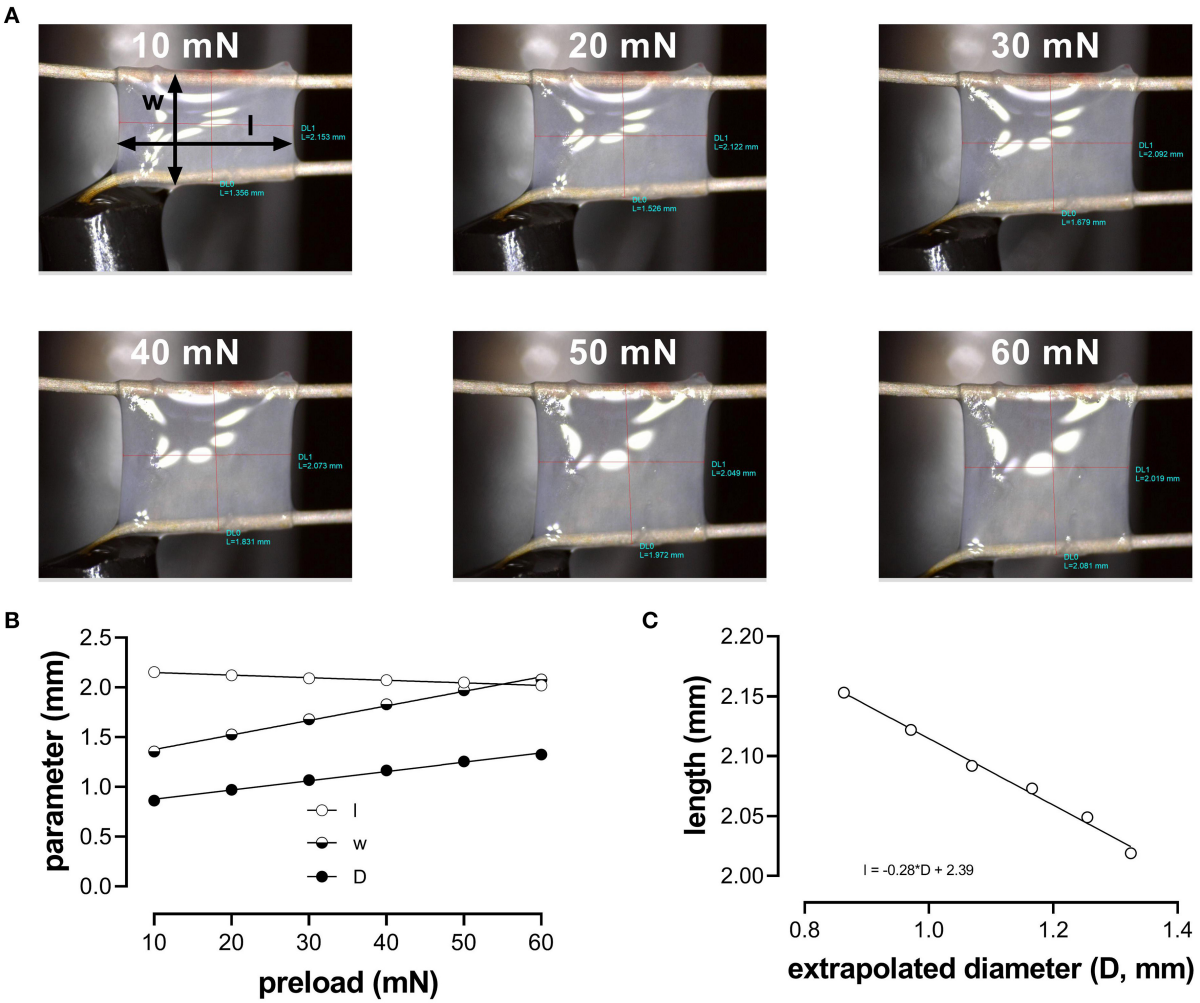
Determination of the aortic segment extrapolated length and diameter. **(A)** Images of the aortic vessel segment at six different preloads between 10 and 60 mN; **(B)** length (l), width (w), and extrapolated diameter (D) as a function of preload; **(C)** relationship between length and extrapolated diameter (to obtain length at each calculated diameter to measure pressure).

Aortic segments were mounted between two parallel wire hooks in 10-ml organ baths. Force and displacement of the upper hook were measured and acquired at 0.4 kHz with a force-length transducer connected to a data acquisition system (Powerlab 8/30 and LabChart 8, ADInstruments). Because the segment is stretched between the two hooks, the extrapolated diameter of the vessel segment (D) at a given preload had to be derived from the displacement of the upper hook, being directly proportional to the inner circumference (Figure 1): $D=2^{*}\frac{w}{\pi }$ with w (width) being the outer distance between the hooks (to approximate the inner circumference of the vessel segment) (see Figure 1A, 10 mN). Before each experiment, width (w) and length (l) were determined at six different preloads (10, 20, 30, 40, 50, and 60 mN), using a stereomicroscope and calibrated image software (Figure 1). To correct for the decrease in segment length with increased width and/or diameter (Figure 1B), the average length of the segment at each cycle (100 ms) was continuously derived from the diameter (D)-length (l) relationship using basic linear regression (Figure 1C).

To estimate the transmural pressure that would exist in the equilibrated vessel segment with the given distension force and dimensions, the following relationship was used: $P=\frac{F}{l^{*}D}$ with F being the force (preload); l, the length as derived from the linear regression in Figure 1B (~2 mm); and D, the diameter of the vessel segment as derived from the displacement of the upper hook. Force was measured directly by the transducer. To simulate cyclic stretch of the segment between two theoretical transmural pressure levels, the preload was adjusted until the desired estimated diastolic and systolic pressure. Although we did not directly measure pressure in the ROTSAC setup but used diastolic and systolic preloads to calculate extrapolated pressures, we decided to use the term “pressure” to indicate “pressure equivalent load”. At all pressures, a stretch amplitude corresponding to 40 mmHg was chosen to allow calculation of compliance and Peterson modulus. Compliance (C) was calculated as follows: $C=\frac{\Delta D}{\Delta P}$ with ΔD being the difference between systolic and diastolic diameter and ΔP being the pressure difference (i.e., 40 mm Hg). The Peterson modulus of elasticity (E_p_) is a frequently used, vessel-size-independent measure of arterial stiffness (Gosling and Budge, 2003) and was calculated as follows: $E_{p}=\;D_{0}*\frac{\Delta P}{\Delta D}$ with D_0_ being the diastolic diameter. During all the experiments, the segments were continuously stretched directly after mounting them in the organ bath with a frequency of 10 Hz to mimic the physiological heart rate in mice (600 bpm) and at an estimated physiological pressure (diastolic 80-systolic 120 mmHg). At ~60 min after isolation of the aorta from the animal, the experiments could be started.

### Experimental Protocols

**Figure 2:**
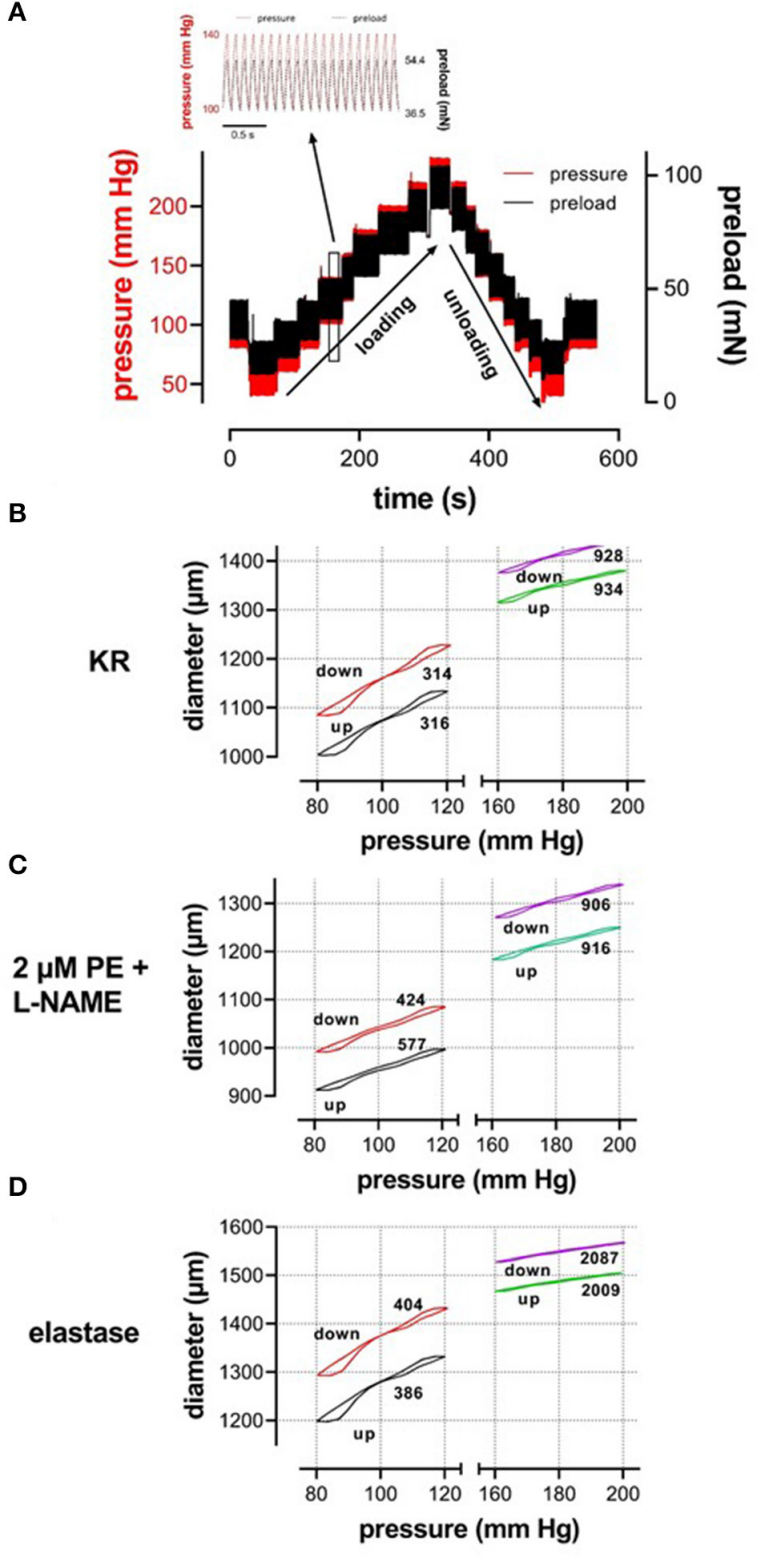
**(A)** Test protocol applied to ROTSAC 1 to measure diameter, compliance, and Peterson modulus, E_p_. Starting at diastolic and systolic preloads, according to 80 and 120 mm Hg, the preloads were adapted to obtain diastolic and systolic pressures of 40 and 80 mm Hg and subsequently increased by 20 mm Hg up to 200–240 mm Hg or higher (loading). Thereafter, the pressure was decreased back to 40–80 mm Hg (unloading) and, finally, to 80–120 mm Hg. In the insert, preload and calculated pressure are shown during the clamp of the segment at 10 Hz between 100 mm Hg (36.5 mN) and 140 mm Hg (54.4 mN). Similar protocols were applied to ROTSAC 2, 3, and 4 with the same pressure steps but other preloads. **(B–D)** Illustrate stretch-by-stretch diameter-pressure loops at 80–120 (black, red) and 160–200 (green, purple) during loading (up, black, green) and unloading (down, red, purple) in three experimental conditions: Krebs-Ringer (KR, **B**), 2 μM PE + 300-μM L-NAME **(C)**, and 0.1 mg/ml elastase **(D)**. The left y-axis refers to diastolic and systolic diameters at 80–120 mm Hg and the right axis to these parameters at 160–200 mm Hg. The y-axis scale is the same in all figures. The numbers along each loop indicate the E_p_ value in mm Hg.

Our setup allows studying aortic stiffness in “isobaric” conditions when pressure is built up, which we have defined as the “loading” protocol or when pressure is released, which we have defined as the “unloading” protocol. These protocols were performed in various experimental conditions. By superimposing the slower loading/unloading protocols on top of the cyclic pulsation, we were able to continuously measure E_p_, as a measure of aortic stiffness, at all applied pressure steps and in any experimental condition. In the present study, all measurements were performed over a broad pressure range (example of the loading/unloading protocols in Figure 2). The stretch amplitude always corresponded to 40 mmHg over the entire pressure range, which means that segment compliance had the same pressure dependency as diameter change but 40 times smaller. It took ~5 min to acquire measurements over the entire pressure range (pressure up direction, loading), and, again, 5 min to return from the highest pressure back to the low pressure (40–80 mm Hg, pressure down direction, unloading). As mentioned before, at any given pressure, diameter-pressure (preload) loops as shown in Figures 2B–D could be determined. It is evident that the slope of the loop decreased considerably with higher pressure (right vs. left part of Figure 2B), with the addition of 2 μM PE in the presence of L-NAME (Figure 2C) and with the digestion of elastin with elastase (Figure 2D). This is reflected by the higher E_p_ values as indicated in each figure. Since diameter loops for single stretch cycles were not the topic of this research, the present study focused on the diastolic and systolic diameters, compliance, and stiffness parameter, E_p_, along the entire pressure range during loading and unloading protocols (Figure 2A). At each pressure range, starting at 40–80 mm Hg and ending at 200–240 mm Hg, diastolic/systolic diameters, compliance, and E_p_ were measured as the median value for the last 20–30 cycles before increasing or decreasing to the next pressure range.

### Experimental Conditions

Group 1 mice: aortic segments (four per mouse) were subjected to cyclic stretch at increasing and decreasing pressure in KR solution. Because the effect of L-NAME was irreversible, subsequently, two segments were treated with 300-μM NΩ-nitro-l-arginine methyl ester (L-NAME) (Sigma-Aldrich, Belgium) to inhibit endothelial nitric oxide synthase (eNOS), followed by the loading/unloading protocols, and two segments did not receive L-NAME. Then, all segments received 2 μM PE, and the loading/unloading protocols were repeated. Since, in the experiments of group 1 mice, two segments received the same protocol, the mean of the data of the two segments was considered for further evaluation.

Groups 2 and 3 mice: aortic segments followed the same protocol as group 1 mouse segments. Young (group 2) and old (group 3) mouse aortic segments were studied separately but received the same experimental protocol. In the last loading/unloading protocol, two segments (one with and one without L-NAME) received 2 μM PE, and the other two segments (again one with and one without L-NAME) received the high extracellular (50 mM) K^+^ concentration.

Group 4 mice: the loading/unloading protocol was applied to aortic segments in KR for all segments in the presence of L-NAME, after contraction with 2 μM PE, following the addition of 0.05 units elastase (ROTSAC 1), 0.1 units/ml elastase (ROTSAC 2), 0.1 mg/ml collagenase (ROTSAC 3), and 0.2 mg/ml collagenase (ROTSAC 4), and, finally, following the addition of 2 μM PE to all organ chambers. Only results for 0.1 units/ml elastase are shown in the present study. The data for 0.05 units/ml elastase and the data for collagenase showed effects on the E_p_-pressure relationships, which were dependent on the enzyme activity and which will be the subject of a follow-up submission.

After each pressure curve, the organ chambers were thoroughly flushed with fresh KR solution. To obtain the 50-mM K^+^ solution, Na^+^ in the KR solution was isosmotically replaced by K^+^. All loading/unloading protocols and measurements were done on “steady state” contractions, achieved 30 min after the addition of PE or high K^+^ to the organ chamber (Fransen et al., 2015). Because the present study was mainly interested in the role of smooth muscle cells in modulating stiffness, we mainly focused on segments in which basal NO release was inhibited by L-NAME and which can be considered as the control (KR) E_p_-pressure relationship. Although E_p_ at 80–120 mm Hg was 299 ± 4 mm Hg and significantly rose to 328 ± 8 mm Hg (*n* = 8, *p* < 0.05) after the addition of 300 μM L-NAME, E_p_ pressure relationships were not statistically different between KR and KR + L-NAME (see Supplementary Figure 1).

Elastase (Elastase suspension, 26. mg P/ml, activity: 4.81 units/mg, Worthington, OH, USA) and collagenase (Collagenase type 2,310 units/mg, Worthington, OH, USA) were applied to the organ chambers for 30 min, while segments were stretched between 80 and 120 mm Hg. Then, the segments were washed three times with KR solution until the diameter change induced by elastase or collagenase was in steady state; after which, the experiment was continued.

### Statistical Analyses

All results are expressed as the mean ± SEM, with *n* representing the number of mice, and analyses were performed using Prism 9.0 (GraphPad Software, La Jolla, CA, USA). The effects of VSMC contraction, pressure, and age on the measured vessel parameters were assessed using a repeated measures (RM) two-way or a three-way ANOVA, if appropriate. Sidak's *post-hoc* test was used to correct for multiple comparisons. Multiplicity-adjusted (when appropriate) *p*-values are reported in the figures and figure legends, a (family-wise) significance level of 5% was selected.

## Results

### Diastolic and Systolic Diameter, Aortic Compliance, and Stiffness Are Pressure and Contraction Dependent

**Figure 3:**
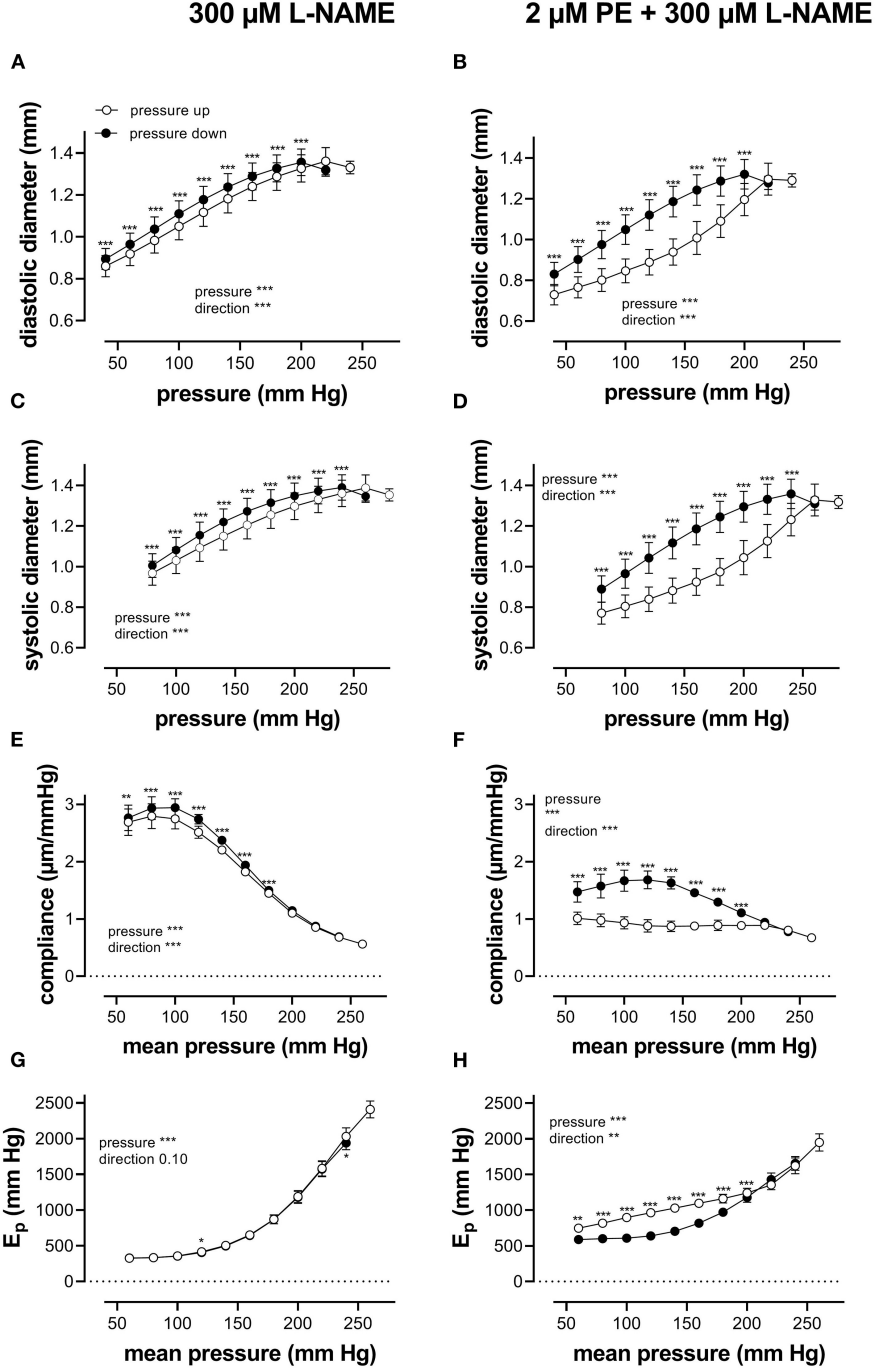
Aortic segments displayed considerable pressure- and contraction-dependent hysteresis. Aortic segments were treated with 300-μM L-NAME in the absence **(A,C,E,G)** and presence **(B,D,F,H)** of 2 μM PE and subjected to cyclic stretch with an amplitude of 40 mm Hg, ranging from 40–80 mm Hg to 240–280 mm Hg (upward direction, open symbols, up) and back to 40–80 mm Hg (downward direction, closed symbols, down). Diastolic and systolic diameters **(A–D)**, compliance **(E,F)**, and Peterson's elastic modulus (E_p_, **G,H**) were determined in aortic segments of 7 C57Bl6 mice (data points ± SEM). Data points for the upward pressure steps (open symbols, up) were compared at any given pressure with data points for the downward pressure steps (closed symbols, down) with two-way RM ANOVA with Sidak's multiple comparison test. ^*^p < 0.05, ^**^p < 0.01, and ^***^p < 0.001.

When repetitively stretched between 80 and 120 mm Hg, aortic segments of group 1 mice (*n* = 7) were stretched by 11 ± 0.2% and stretch slope of 2.38 ± 0.003 μm/mm Hg (*n* = 7), which is in accordance with normal stretch of 10% in the human aorta [13, 14]. As expected, extrapolated and calculated diastolic and systolic diameters were strongly dependent on the distension pressure. In baseline conditions after inhibition of basal NO release with 300 μM L-NAME (Figures 3A,C,E,G), the diastolic and systolic diameters of the aortic diameters increased with pressure. Diameters increased with pressure starting at 40–80 mm Hg up to 240–280 mm Hg (up) until a “maximum” diameter was attained of 1.36 ± 0.07 mm (*n* = 7) for diastolic pressure at 220 mm Hg and 1.39 ± 0.07 mm (*n* = 7) for systolic pressure at 260 mm Hg. Upon lowering diastolic and systolic pressure back down to 40–80 mm Hg (down, unloading), both diastolic and systolic diameters decreased but were significantly larger than during the pressure increase, indicating hysteresis. Diameter change between diastolic and systolic pressure divided by the pulse pressure (40 mm Hg) revealed the compliance (in μm/mm Hg) (Figure 3E). Compliance measured during loading was significantly lower than for unloading in the pressure range below 160 mm Hg (140–180 mm Hg) but was similar for higher pressures. Finally, the aortic stiffness parameter, E_p_, did not show significant hysteresis in baseline conditions (Figure 3G), and, at any given pressure, E_p_, upon loading, was not different from E_p_ upon unloading.

Diameters, compliance, and Peterson modulus, E_p_, were also determined in the presence of 2 μM PE at gradually increasing and decreasing pressures (Figures 3B,D,F,H) and the following depolarization with 50-mM extracellular K^+^ (data not shown). After the addition of 2 μM PE in the presence of L-NAME, hysteresis was considerably increased for all parameters. As expected, diastolic and systolic diameters decrease with the addition of PE and after attaining maximal values of 1.30 ± 0.08 mm and 1.33 ± 0.08 mm (*n* = 6) at 240 and 280 mm Hg, respectively, diameters for the downward pressure steps were, at any given pressure, significantly increased when compared with the upward pressure steps (Figures 3B,D). Compliance (Figure 3F) was nearly three times lower when compared with control conditions (Figure 3E) and was almost pressure-independent for the upward pressure steps. Unloading resulted in clear pressure dependency but was not different from loading compliance for pressures above 220 mm Hg. Similar observations apply for E_p_ with significantly lower E_p_ values at any pressure below 220 mm Hg during unloading. We defined these lower stiffness values as “de-stiffening” of the aortic segments for the downward pressure steps below 220 mm Hg (Figure 3H). For depolarization with 50 mM K^+^, similar results were obtained (see also **Figure 6**). Finally, it should be mentioned that when segments in the presence of PE and L-NAME were stretched at 80–120 mm Hg after the pressure protocol, it took about 20 to 30 min to attain E_p_ values comparable with E_p_ before the pressure protocol (see Supplementary Figure 2). Only after this “recovery” or “re-stiffening” period, another experimental protocol could be performed. These data suggest that the contractile state of vascular smooth muscle cells determines the hysteresis phenomena described in Figure 3 and that at any given pressure, stiffness in unloading conditions is lower than stiffness in loading conditions, which we called de-stiffening.

### Age and Contractile Stimulus Affect Aortic Stiffness

**Figure 4:**
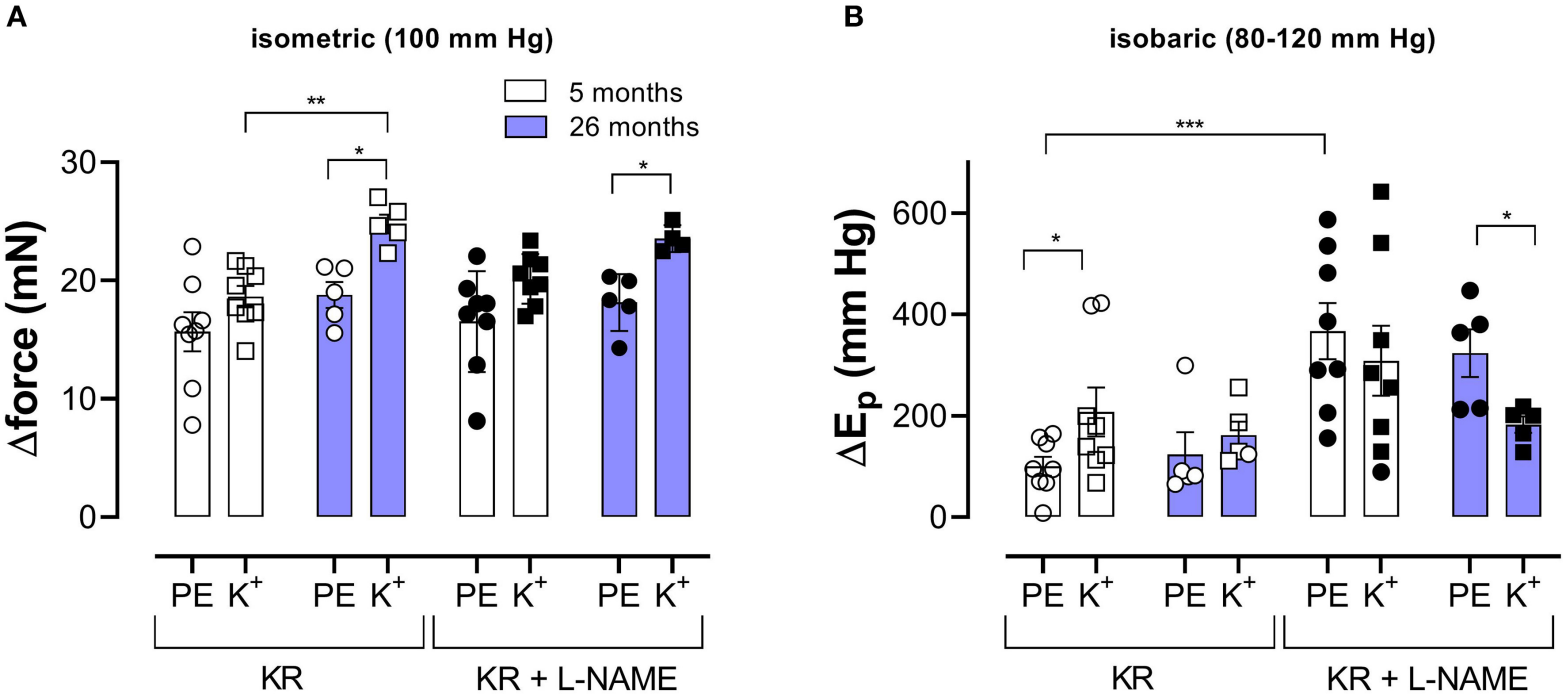
α_1_-Adrenergic stimulation and maximal depolarization of aortic segments cause different isometric and isobaric effects. Aortic segments were mounted in isometric (100 mm Hg, **A**) and isobaric (80–120 mm Hg, **B**) conditions and treated with 2 μM PE (circles) or depolarized with 50 mM K^+^ (squares) in the absence (open symbols) and presence (closed symbols) of 300-μM L-NAME. The increase of force (Δorce) and E_p_ (ΔE_p_) was determined in aortic segments of 5 (white bars) and 26 (blue bars) months old mice. Two-way RM ANOVA with Sidak's multiple comparison test. ^*^p < 0.05, ^**^p < 0.01, and ^***^p < 0.001.

Pressure- and contraction-dependent hysteresis, as described above, was further studied in aortic segments of young (5 months, group 2, *n* = 8) and old (26 months, group 3, *n* = 5) mice. Contraction was not only induced by α_1_-adrenergic stimulation with 2 μM PE but also by receptor-independent depolarization by 50 mM K^+^ in the absence and presence of 300-μM L-NAME to inhibit basal endothelial NO release. Figure 4 compares the effects of 2 μM PE and 50 mM K^+^ before and after inhibition of basal NO release with 300-μM L-NAME in aortic segments of 5- and 26-month-old mice mounted in static, isometric conditions (100 mm Hg, Figure 4A) and dynamic, isobaric conditions (80–120 mm Hg, Figure 4B). The figure shows that maximal isometric force was higher after depolarization than following α_1_ adrenoceptor stimulation, especially in aged mice but was not significantly age-dependent. In isobaric conditions, aortic stiffness was significantly increased by addition of L-NAME for PE-induced E_p_ increase in young animals. In the presence of L-NAME, E_p_ increased more after α_1_ adrenoceptor stimulation than following depolarization with high K^+^ in aged animals. Also, here, there was no significant age-dependent effect.

### Pressure-Dependent Hysteresis Is Contraction- and Age-Dependent

**Figure 5:**
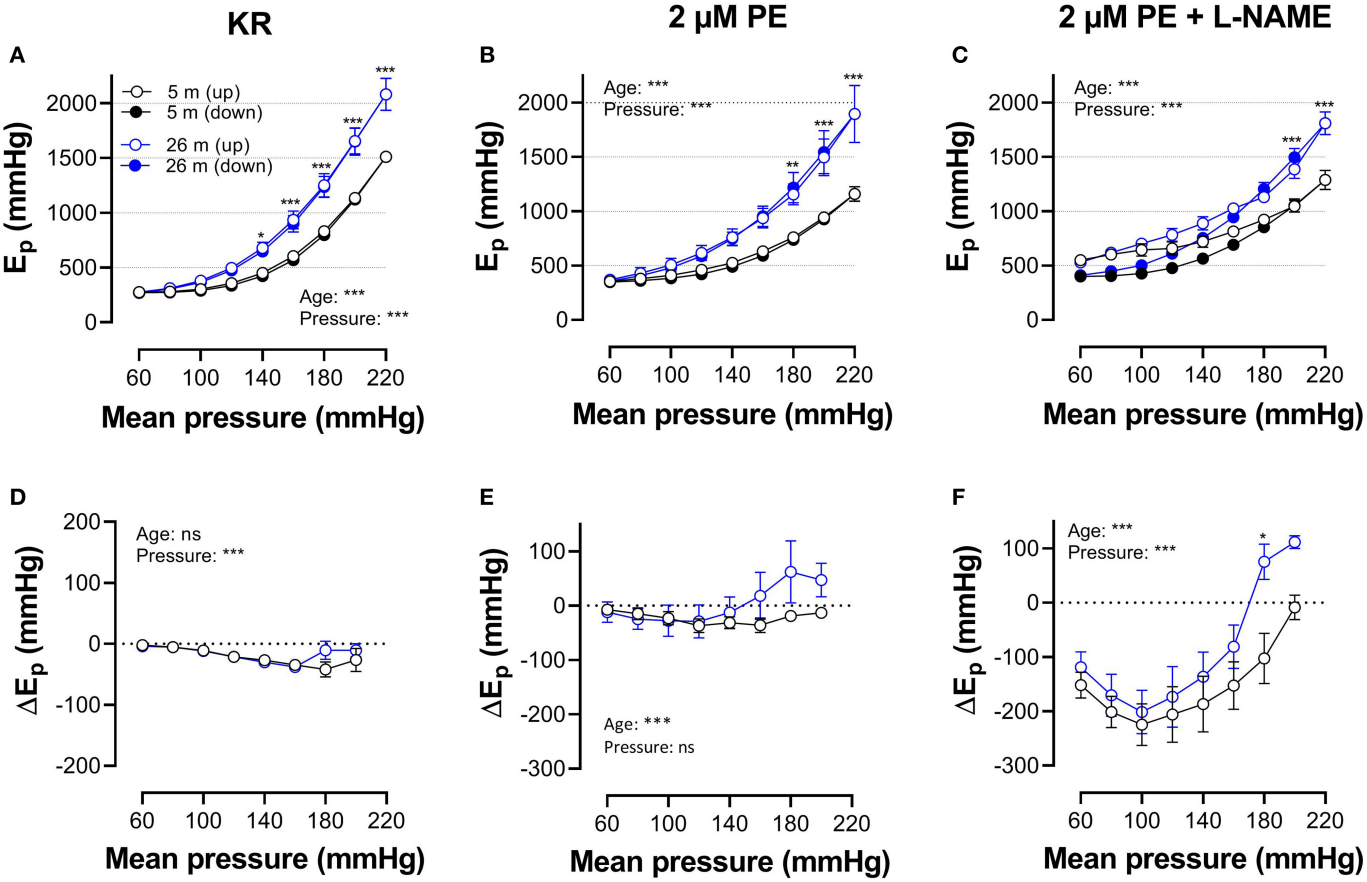
Hysteresis is pressure, contraction and age dependent. Aortic segments of mice aged 5 months (black, *n* = 8) and 26 months (blue, *n* = 5) were subjected to upward (open symbols, up) and downward (closed symbols, down) pressure steps between 40 and 80 mm Hg to 200–240 mm Hg and back in control conditions (KR, **A,D**) in the presence of 2 μM PE **(B,E)** and 2 μM PE in the presence of 300-μM L-NAME to block endothelial NO release **(C,F)**. Absolute values of E_p_ are shown in **(A–C)**, whereas, in **(D–F)**, data points for the upward pressure steps (open symbols, up) were compared at any given pressure, with data points for the downward pressure steps (closed symbols, down) (stiffness hysteresis). Two-way RM ANOVA with Sidak's multiple comparison test, ^*^p < 0.05, ^**^p < 0.01, ^***^p < 0.001.

**Figure 6:**
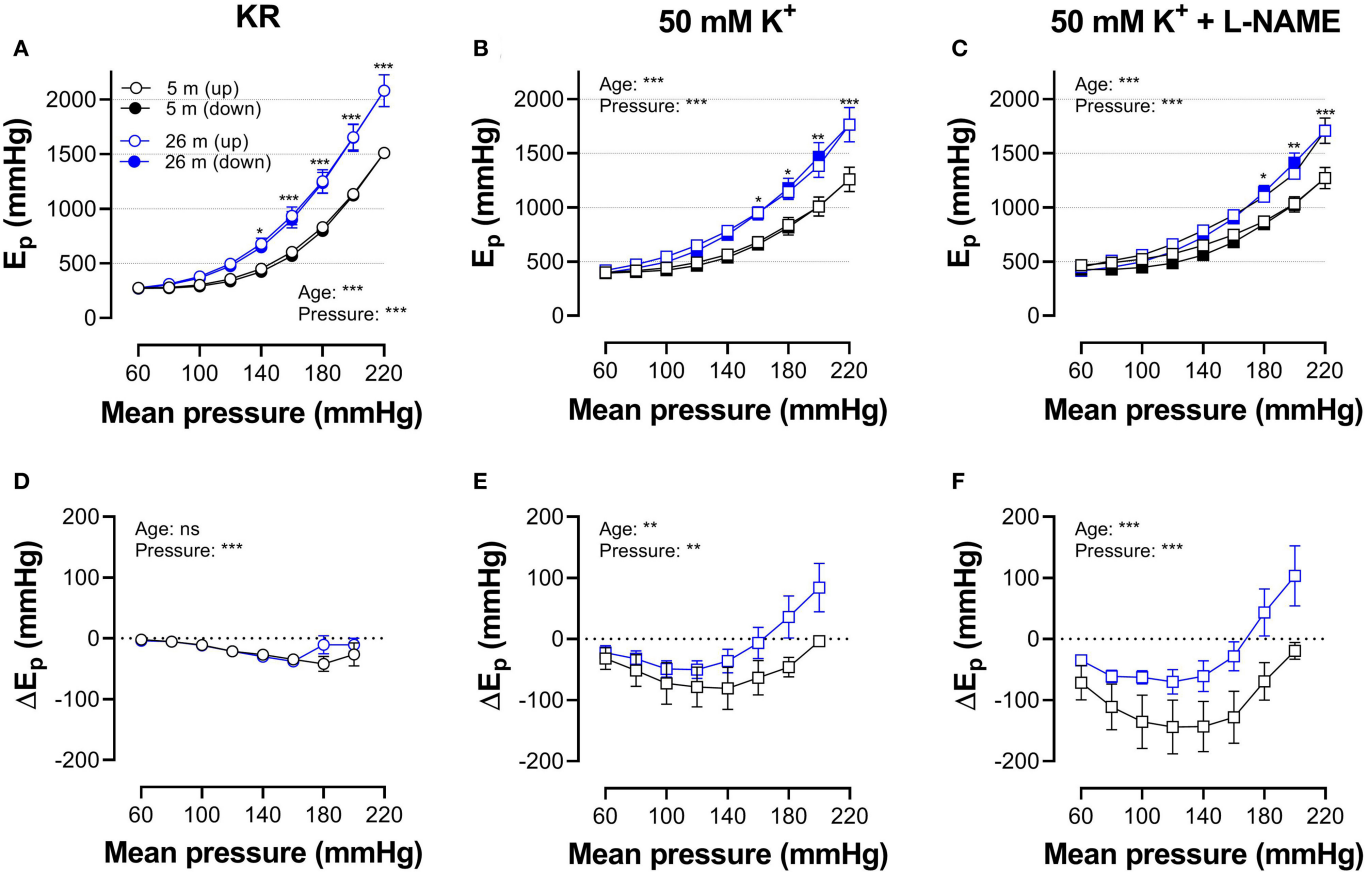
Hysteresis is pressure, contraction, and age dependent. Aortic segments of mice aged 5 months (black, *n* = 8) and 26 months (blue, *n* = 5) were subjected to upward (open symbols, up) and downward (closed symbols, down) pressure steps between 40 and 80 mm Hg to 180–220 or 200–240 mm Hg and back in control conditions (KR, **A,D**) in the presence of 50 mM K^+^ **(B,E)** and 50 mMK^+^in the presence of 300-μM L-NAME to block endothelial NO release **(C,F)**. Absolute values of E_p_ are shown in **(A–C)**, whereas, in **(D–F)**, data points for the upward pressure steps (open symbols, up) were compared at any given pressure with data points for the downward pressure steps (closed symbols, down) (stiffness hysteresis). Two-way RM ANOVA with Sidak's multiple comparison test (old vs. young mice), ^*^p < 0.05, ^**^p < 0.01, ^***^p < 0.001.

Figures 5, 6 show E_p_ values for up- and downward pressure steps in the presence of 2 μM PE and 50 mM K^+^ in the absence and presence of 300-μM L-NAME for aortic segments of young and old mice. E_p_ in control conditions (KR) was age- and pressure-dependent (Figures 5, 6A) with significantly higher stiffness at elevated distension pressure for aortic segments of old mice. This age-dependent difference in aortic stiffness remained, following stimulating contraction with 2 μM PE or 50 mM K^+^ in the absence (Figures 5B, 6B) or presence (Figures 5C, 6C) of L-NAME. Both contractile stimuli increase E_p_ at low and decrease E_p_ at high-distension pressures, especially in aortic segments of young mice. By subtracting the E_p_ values for the downward pressure steps from E_p_ values for the corresponding upward pressure steps, hysteresis is visualized as a pressure-dependent difference in E_p_ (ΔEp, Figures 5D–F, 6D–F) with negative values corresponding to lower stiffness or “de-stiffening.” In the absence of contractile stimuli, hysteresis was nearly absent. Following contraction of the aortic segments, hysteresis led to lower stiffness (negative values for △Ep) along the whole pressure range for aortic segments of young mice and was more evident in the absence of basal NO release (presence of 300-μM L-NAME) (Figures 5F, 6F). In aortic segments of old mice, hysteresis in contracted segments was smaller for both contractile stimuli. Moreover, in these segments, lower stiffness in the pressure range from 40–80 up to 160–200 mm Hg turned to higher stiffness in the pressure range above 160–200 mm Hg.

**Figure 7:**
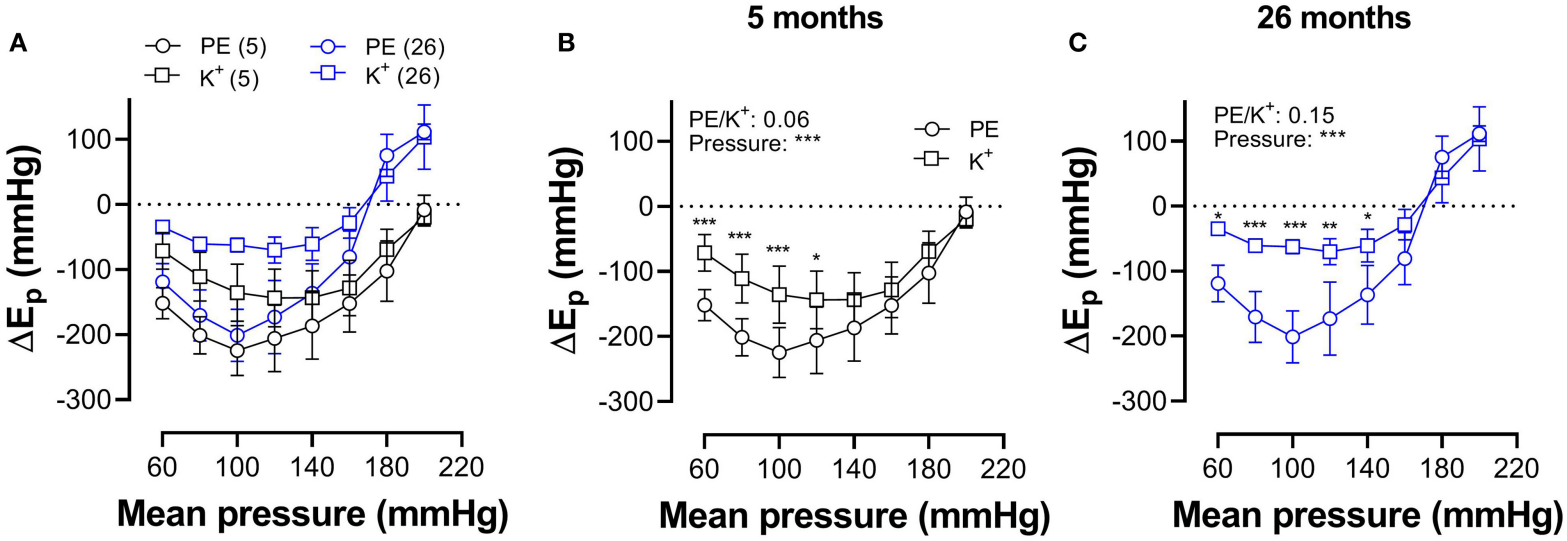
Pressure-dependent hysteresis is affected by the contractile stimulus and by age. Data from Figures 5F, 6F are combined in Figure 6A for mice of 5 months (*n* = 8, 5, black) and 26 months (*n* = 5, 26, blue). Contractions by 50 mM K^+^ (squares) and 2 μM PE (circles) were elicited in the presence of L-NAME. Three-way ANOVA: pressure: ^***^; age 0.12; stimulus ^*^; pressure/age: ^**^; pressure/stimulus ^***^; age/stimulus 0.73. For clarity, **(A)** was repeated in **(B)** (for 5 months) and **(C)** (for 26 months). Two-way RM ANOVA with Sidak's multiple comparison test, ^*^p < 0.05, ^**^p < 0.01, ^***^p < 0.001.

In Figure 7, which reflects the same data as in Figures 5, 6, hysteresis in young and old mice is compared for maximal contractions (NO release inhibited with L-NAME) induced by depolarization and α_1_-adrenergic stimulation.

In young animals, hysteresis induced by contraction with PE or depolarization resulted in lower stiffness upon unloading along the whole pressure range up to 200 mm Hg, whereas, in old mice, hysteresis changed from lower to higher stiffness upon unloading at pressures above 160 mm Hg. Hysteresis by depolarization was significantly smaller in magnitude than hysteresis by PE, especially in the lower pressure range and for old animals.

### Elastase Attenuates Pressure- and Contraction-Dependent Hysteresis

**Figure 8:**
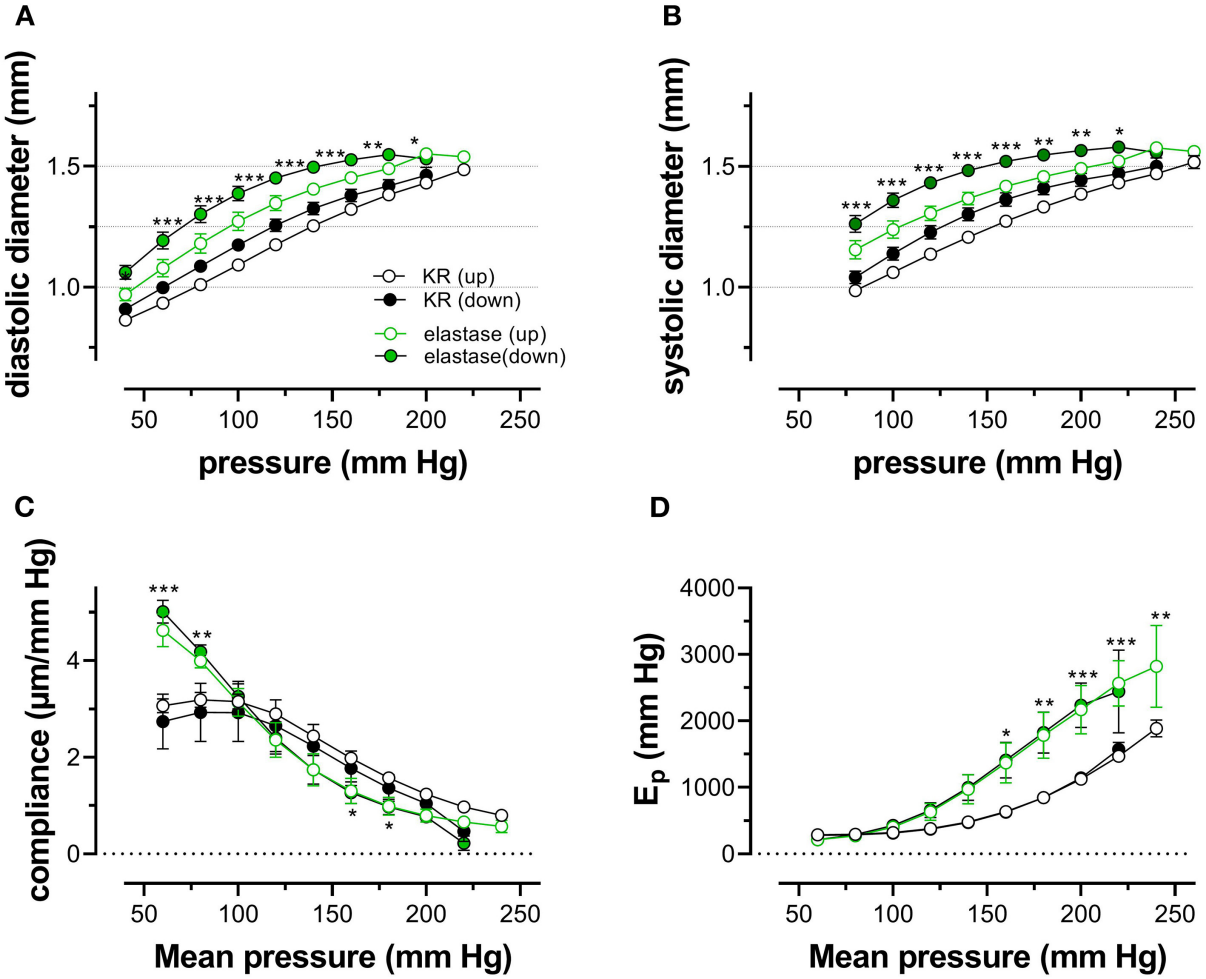
Diastolic and systolic diameters, compliance, and stiffness are elastin dependent. Diastolic diameter **(A)**, systolic diameter **(B)**, compliance **(C)**, and Peterson modulus, E_p_ **(D)**, were measured in aortic segments in control conditions (black symbols) or after treatment with 0.1 unit/ml elastase (green symbols). Data points for the upward pressure steps (open symbols, up) were compared at any given pressure with data points for the downward pressure steps (closed symbols, down). Two-way RM ANOVA with Sidak's multiple comparison test (elastase vs. control), ^*^p < 0.05, ^**^p < 0.01, ^***^p < 0.001, *n* = 5.

Because one of the characteristics of vascular aging is the age-dependent breakdown of elastin, aortic segments of group 4 mice were treated with 0.1 unit/ml elastase. In baseline conditions, the breakdown of elastin caused a significant increase of diastolic and systolic diameters (Figures 8A,B), especially at physiological pressures. It is evident that at, high-distension pressure, diastolic and systolic diameters, after elastase treatment, are nearly the same. Hence, the compliance, which is defined as the change of diameter per mm Hg distension pressure, is approaching zero at high pressures (Figure 8C). At very low pressure (<100 mm Hg), compliance was higher than in control conditions and rapidly fell to below control levels at pressures above 100 mm Hg. Elastase-treated aortas were significantly stiffer than control aortas at pressures above 140 mm Hg (Figure 8D).

**Figure 9:**
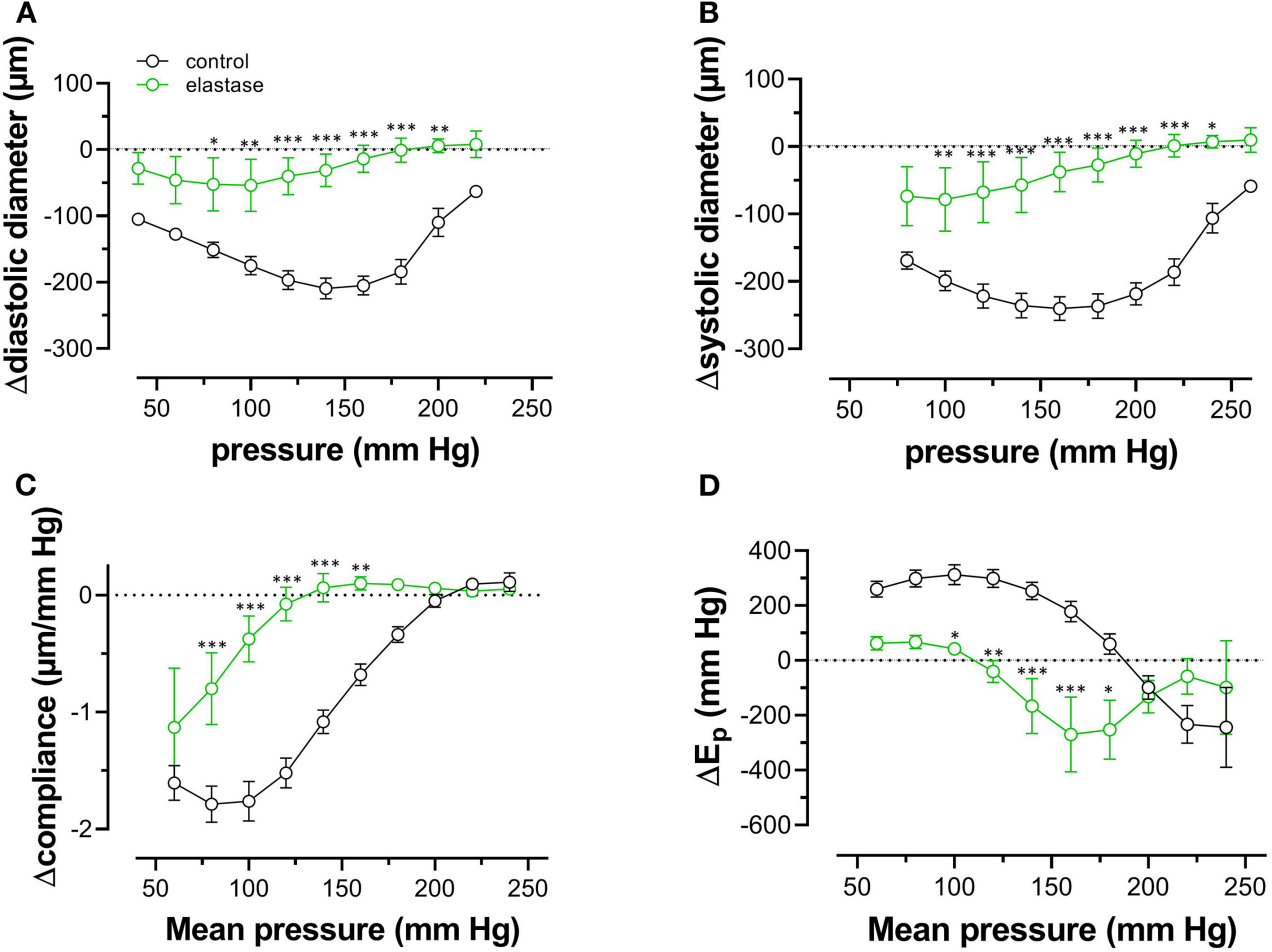
Diastolic and systolic diameters, compliance, and stiffness in the presence of PE are elastin dependent. Diastolic diameter **(A)**, systolic diameter **(B)**, compliance **(C)**, and Peterson modulus, E_p_ **(D)**, were measured in aortic segments in the presence of 2 μM PE and 300-μM L-NAME in control conditions (black symbols) or after treatment with 0.1 unit/ml elastase (green symbols). Two-way RM ANOVA with Sidak's multiple comparison test (elastase vs. control), ^*^p < 0.05, ^**^p < 0.01, ^***^p < 0.001, *n* = 5.

Diameters, compliance, and E_p_ were determined also in the presence of 2 μM PE and 300-μM L-NAME. To reveal the effects of PE in the absence and presence of elastase, the difference between the biomechanical parameters in the absence and presence of PE is displayed in Figure 9. In the absence of elastase, PE caused, as expected, decrease of diastolic and systolic diameters, reduced compliance, and elevated aortic stiffness. All these parameters were pressure-dependent, and PE was less effective at high-distension pressures (Leloup et al., 2019), and the pressure at which the PE effect caused higher compliance and lower E_p_ was ~180–200 mm Hg. After degradation of elastin with elastase, all effects of PE were significantly attenuated in the physiological pressure range (decreased diameters and compliance, increased E_p_). Furthermore, in the elastase-treated segments, the pressure at which the PE-induced decrease of compliance and increase of E_p_ changed a sign (higher compliance and lower E_p_) and shifted to lower pressures by ±50 mm Hg.

**Figure 10:**
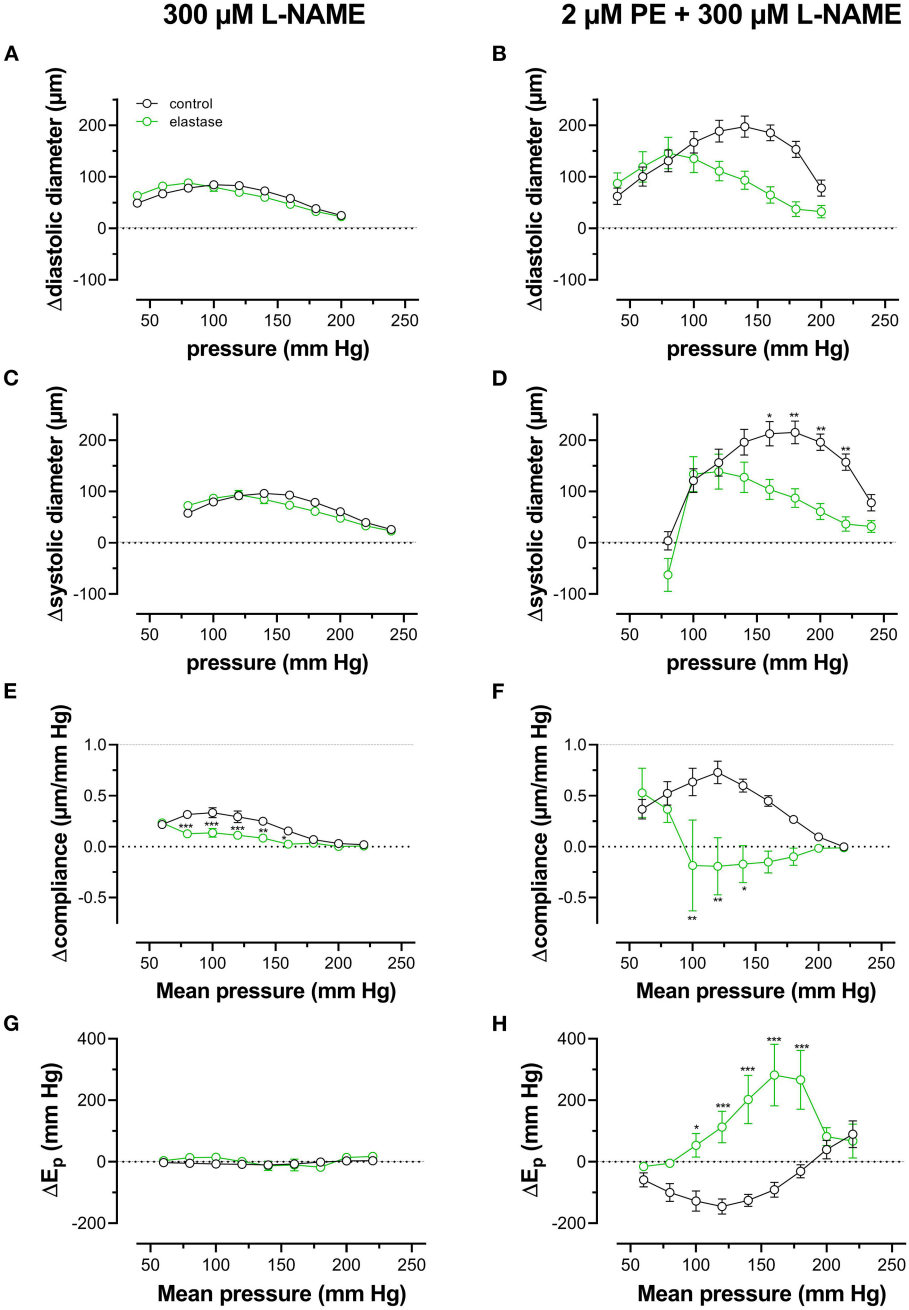
Pressure-dependent hysteresis induced by contraction with PE is elastin dependent. Diastolic **(A,B)** and systolic diameters **(C,D)**, compliance **(E,F)**, and E_p_ **(G,H)** were determined in untreated (black) and elastase-treated (0.1 unit/ml, green) aortic segments in KR **(A,C,E,G)** and in the presence of 2 μM PE/300-μM L-NAME **(B,D,F,H)**. Data points for the upward pressure steps were compared at any given pressure with data points for the downward pressure steps (stiffness hysteresis). Hence, positive values for diameter and compliance refer to larger diameters and compliance for downward pressure steps. Negative values for E_p_ correspond to lower stiffness for the downward pressure steps (“de-stiffening”). Two-way RM ANOVA with Sidak's multiple comparison test, ^*^p < 0.05, ^**^p < 0.01, ^***^p < 0.001, *n* = 5.

Finally, pressure-dependent hysteresis was studied in the absence/presence of 2 μM PE and 300-μM L-NAME and absence /presence of elastase. The Δdiameter, Δcompliance, and ΔE_p_ values between upward and downward pressure steps at any given pressure (Δparameter by hysteresis) are shown in Figure 10. In the absence of contraction, hysteresis was only evident for diameters and compliance, but not for E_p_. Elastase treatment was ineffective in affecting hysteresis for diameters, but compliance hysteresis was significantly attenuated at physiological pressures (Figure 10E). Hysteresis for all parameters was significantly intensified by contraction with PE in elastin-intact segments. Diameters and compliance were significantly increased for downward pressure steps, whereas segments were less stiff when the segments were unloaded at mean pressures below 160 mm Hg (Figures 10B,D,F,H). In segments treated with 0.1 unit/ml elastase, on the other hand, diameter and compliance hysteresis was reduced at pressures above 100 mm Hg. Hysteresis of aortic stiffness was completely reversed with higher stiffness at any given pressure upon unloading (positive ΔE_p_ values in Figure 10H).

## Discussion

The present study revealed that the ROTSAC equipment (Leloup et al., 2016) was suitable to study pressure dependency of arterial stiffness. The pressure-stiffness relationship of the present study displayed key features of anisotropic artery walls with non-linear behavior, being soft at low pressure and increasing stiffness at higher pressures. In this way, they perfectly resemble classical aortic stress-strain relationships (Hong et al., 2015). Moreover, the setup also allowed to study *ex vivo* viscoelastic properties and stiffness hysteresis of isolated mouse aortic segments. Indeed, dynamic testing of arterial stiffness, whether *in vivo* or *ex vivo*, should address viscoelasticity alongside the measurement of stiffness. *In vivo*, acute manipulation (pharmacological or mechanical) of arterial pressure allows to compare arterial stiffness at the same level of pressure in different animals and to investigate the pressure sensitivity of arterial stiffness (Butlin et al., 2020). For example, chronic hypertension has been described to decrease the pressure sensitivity of arterial stiffness (Ng et al., 2012).

### Aortic Stiffness Hysteresis

In the present study, the pressure-diameter relationship for a single-stretch cycle displayed hysteresis with smaller diameters upon increasing than upon decreasing stretch (Figures 2B–D). In general, it is assumed that cell stretch temporarily causes higher stress, followed by a return to the basal level. Hence, cyclic stretch is assumed to cause stress-strain hysteresis, with higher stresses during building up stretch than during subsequent stretch release. This has been demonstrated in numerous studies *in vivo* and *ex vivo* (Bauer et al., 1979; Langewouters et al., 1986; Armentano et al., 1995; Boutouyrie et al., 1997; Trepat et al., 2004) and illustrates the viscoelastic nature of blood vessels. The present study did not focus on hysteresis during a single-stretch cycle but investigated whether aortic stiffness also displayed hysteresis phenomena when segments were loaded and unloaded in a pressure range from 60 to 240 mm Hg, while subjected to continuous cyclic stretch with a constant PP of 40 mm Hg at high frequency. At increasing, and subsequently decreasing pressures, diastolic and systolic diameters, compliance, the aortic stiffness parameter, and Peterson's modulus or E_p_ were compared at any given pressure or mean pressure in the loading and unloading direction. In baseline conditions (KR), diastolic and systolic diameters and compliance for a pulse pressure of 40 mm Hg were smaller at any given pressure upon progressive loading of the segments than upon unloading. Stiffness hysteresis was, however, absent (Figures 3A,C,E,G), mainly because the diameter change in the loading and unloading direction was nearly the same. Hence, aortic stiffness gradually increases with pressure, but the pressure-stiffness relationship was similar in the loading (increasing pressure) and unloading (decreasing pressure) directions.

In general, it is assumed that the passive mechanical behavior of vascular walls is mainly governed by the extracellular matrix (collagen, elastic fibers, proteoglycans, and water), whereas the active mechanical contribution is due to the VSMCs (Holzapfel and Ogden, 2018; Butlin et al., 2020). The interactions between both in regulating aortic compliance are, however, complex and both are definitely crucial elements in the determination of arterial wall stiffness (Saphirstein et al., 2013; Hong et al., 2015; Lacolley et al., 2017). Elastic arteries, such as the aorta, are composed of alternating layers of smooth muscle and elastin-containing fibers. Elastin degradation with elastase caused prominent changes in the pressure-diameters, -compliance, and -stiffness relationships. Vessel diameters were significantly increased, compliance was larger at low pressure (60 mm Hg), but compromised at higher pressures, and E_p_ was significantly increased at pressures of 140 mm Hg. Similarly, the aorta of elastin heterozygous mice (Eln^+/−^), in which about 60% of the normal elastin amount is present (Wagenseil and Mecham, 2009), is stiffer than the aorta of Eln^+/+^ mice (Knutsen et al., 2018). The higher stiffness of Eln^+/−^ or elastase-treated aortas is believed to be due to the redistribution of tensile force to the stiffer collagen. Because aging is accompanied by a gradual increase of elastin breaks and loss of elasticity, this process is also believed to be at the base of progressive arterial stiffening during aging. Also, the present study showed that, in comparison with “young” mice (5 months), aortic segments of old mice (26 months) displayed higher E_p_ values and aortic stiffness at mean pressures above 100 mm Hg.

### Aortic Stiffness Hysteresis Modulation

The presence of a considerable amount of VSMCs in the aorta means that, by generating contractile force, they obviously contribute significantly to the material behavior of the artery wall (Qiu et al., 2010; Poythress et al., 2013; Saphirstein et al., 2013; Gao et al., 2014; Hong et al., 2015; Zhang et al., 2016). Aortic VSMC function can be significantly altered by cyclic stretch (Leloup et al., 2017), and, when subjected to high-frequency (10 Hz) stretch of about 10% in baseline conditions, it is expected that the ECM, together with actomyosin contraction and cytoskeletal remodeling, may produce viscoelastic-like stress relaxation and hysteresis. Thereby, it is assumed that VSMC may contribute markedly to different hysteresis behaviors under different loading regimes (Win et al., 2018).

In the presence of PE and L-NAME, aortic stiffness was almost independent of pressure when pressure was built up [compared with the reduced pressure-sensitivity of stiffness in hypertensive animals (Ng et al., 2012)]. Release of pressure reintroduced “control-like” pressure dependency with maximal compliance again in the physiological pressure range. When compared with baseline conditions, PE caused aortic stiffening in the pressure range between 60 and 180 mm Hg, but, at the highest pressure, stiffness was reduced (Figure 5). This has been explained by a shift in the load-bearing component from collagen at high pressures to the contractile VSMCs at lower pressures (Leloup et al., 2019). In the present study, pressure-induced hysteresis was dependent on the contractile state of the aortic VSMCs. In comparison with baseline conditions, contraction of the aortic VSMCs with α_1_ adrenergic stimulation or with depolarization by high extracellular K^+^, especially after inhibition of basal NO release with L-NAME, caused more pronounced hysteresis of all parameters (Figures 3B,D,F,H). In this condition, now, also, clear hysteresis of the aortic stiffness parameter, E_p_, occurred with lower stiffness (“de-stiffening”) in the unloading than in the loading direction at any pressure given between 60 and 200 mm Hg. Both depolarization with high K^+^ and α_1_ adrenoceptor stimulation with PE caused aortic stiffness hysteresis. Although 50 mM K^+^ caused larger isometric contractions than PE, stiffness increase by depolarization was significantly smaller than by PE (Figure 4). Furthermore, aortic stiffness hysteresis was more pronounced for PE than for 50 mM K^+^. The largest difference between upward and downward pressure change was observed at physiological pressures for PE and slightly higher pressures for depolarization (Figure 7). This is the pressure range where the tensile force is mainly absorbed by the contracted VSMCs, suggesting that aortic VSMCs, indeed, play a major role in aortic stiffening, stiffness hysteresis phenomena, and aortic viscosity. The more pronounced hysteresis for PE-induced stiffening may be explained by the stronger activation of Rho/Rho kinase in receptor-mediated (adrenoceptor, PE) than non-receptor-mediated (high K^+^) contraction. Rho-linked signaling mechanisms have been linked to mechanotransduction *via* modulation of focal adhesions (Jang et al., 2005; Sun et al., 2012). Focal adhesion sites, where the VSMC cytoskeleton is linked to ECM components through integrin-based interactions (Saphirstein et al., 2013; Lacolley et al., 2017), permit adequate force transmission from the contracted VSMC to the vascular wall *via* the extracellular matrix, thereby enabling stiffness development. Pharmacological inhibition of focal adhesions results in a more than 60% decrease in vascular stiffening, highlighting their importance in the VSMC contribution to arterial stiffness (Min et al., 2012; Poythress et al., 2013; Gao et al., 2014).

On the other hand, the continuous connection of the elastin fibers to the contractile units in the VSMCs of the aortic wall, which has been termed the “elastin-contractile unit” (Milewicz et al., 2017), is a prerequisite for performing contraction. The present study confirmed these observations. The effects of PE-induced constriction on diameters, compliance, and E_p_ were reduced by 70 to 100% at low to high pressure. At the high-pressure elastase-treated aortic segment, stiffness was even insensitive to the presence of PE. The “elastin-contractile unit” (Milewicz et al., 2017) may also affect the viscoelastic properties and hysteresis of the aorta. In aortic segments treated with elastase and subjected to PE in the presence of L-NAME, degradation of elastin caused hysteresis, which was exactly the opposite of stiffness hysteresis in elastin-intact aortic segments. At any given mean pressure in the unloading conditions, aortic stiffness was higher than in the loading condition (Figure 10). This “reverse” hysteresis was also observed in single VSMCs exposed to stretch transverse to primary fiber alignment (Win et al., 2018), suggesting that rearrangement of the ECM occurred after elastin digestion in our experiments may affect viscous properties of the aortic segments.

Aging and VSMC stimulation with depolarization or α_1_ adrenoceptor stimulation changed the typical biomechanical behavior of the aorta. The most prominent effect of aging was the increased aortic stiffness along the entire pressure range investigated. Especially at higher-distension pressure, aortic stiffness was increased. Moreover, stiffness hysteresis in the aorta of old mice was of a mixed type. Stiffness at mean pressures above 160 mm Hg was higher for pressure release than for pressure increase. Below 160 mm Hg, normal hysteresis was present with stiffness being lower in the unloading than loading direction. Aorta segments of young mice showed normal stiffness hysteresis at mean pressures below 200 mm Hg, about 40 mm Hg above the turning point in aortas of old animals. It is well-known that aging causes migration of VSMCs from the media to the intima, wall thickening, and artery stiffening because of the fragmentation and degradation of elastin, cross-linking, collagen remodeling, and ECM irregularities (Toda et al., 1980; Greenwald, 2007; Tsamis et al., 2013). The fact that elastin degradation with elastase turned normal pressure-dependent hysteresis of contracted aortic segments to “reverse” hysteresis may indicate that the mixed hysteresis observed in aortic segments of old mice is due to the age-dependent elastin degradation and/or the elevated number of elastin breaks. However, many different structural and biochemical changes occur within the vessel wall with aging (Ungvari et al., 2018), and recent studies have shown an increase in VSMC cellular stiffness with aging (Qiu et al., 2010). Aging is characterized by an increase in α smooth muscle actin, the stress fiber-specific isoform for VSMCs, which is central to mechanotransduction by transmitting force to the ECM *via* integrins (Qiu et al., 2010). Hence, also, with aging, focal adhesion assembly and disassembly may be affected. In aortas of old mice, the inhibitory effects of the Src inhibitor PP2 on agonist-induced stress, stiffness, and phosphotyrosine increase are lost, suggesting the regulatory pathways related to focal adhesion are attenuated with aging (Gao et al., 2014).

### Physiological Impact, Shortcomings, and Future Directions of This Study

Because of its distinguished location between the contracting and ejecting left ventricle of the heart and the rest of the circulation, the compliance and stiffness of the aorta play a pivotal role in cardiovascular health. The elastic properties of the aorta undoubtedly contribute to the blood buffering capacity of this blood vessel, as well as its viscous properties in the dynamic regulation of wall properties. Results of the present study indicate that both ECM and VSMCs contribute to the pressure-dependent stiffness hysteresis observed as the difference between loading and unloading stiffness along a pressure range from 60 to 240 mm Hg. To investigate this in more detail, more experiments with elastase, collagenase, and factors involved in focal adhesion dynamics are expected to further elucidate the pressure-, age- and contraction-dependent biomechanics of the aorta. An important physiological aspect of the viscous properties of the aortic wall might be a de-stiffening effect of acute exercise. During exercise, diastolic pressure hardly changes, whereas systolic pressure is elevated dependent on the exercise intensity, leading to an increase of pulse pressure. The results of the present study suggest that immediately after an exercise bout, diastolic and systolic diameters are larger than before exercise and dependent on the contractile state of the VSMCs; this may lead to aortic de-stiffening and a higher blood buffering capacity of the aorta immediately after the exercise bout.

One of the main shortcomings of this study is that aortic segments were not pressurized and were only subjected to uniaxial stretch. *Ex vivo* organ bath studies on isolated aortic segments have been performed extensively in the past few decades. In many cases, researchers focused on the isometric force at non-physiological preloads or stress-strain relationships under static conditions. Recently, a new setup to study biaxial biomechanics of mouse carotid artery under pulsatile conditions has been described (van der Bruggen et al., 2021). With this setup, it was demonstrated that pulse wave velocity, distensibility, and compliance coefficients of murine carotid artery depended on the amount of axial stretch. Although our ROTSAC system uses uniaxial stretch of large mouse aortic segments (diameter ± 1 mm) or larger, it allows to continuously stretch aortic segments at high frequency of 10 Hz (600 beats/min) or even more and to apply extrapolated pressure differences of up to 100 mm Hg. In this way, the ROTSAC setup allows simulating various pulsatile conditions or bouts of exercise. In the ROTSAC setup, compliance of the segments was defined as the change in diameter (between systolic and diastolic pressures) per mm Hg pressure. In general, compliance refers to the change of volume per mm Hg pressure. We preferred to keep the change of diameter per mm Hg, because diameters were directly measured and extrapolated in the present setup. Also, wall thickness was not measured and considered. Therefore, we were not able to measure wall stress. It should be mentioned that, to apply a calculated pressure of 100 mm Hg to the aortic segments, it was found that aortic segments of young mice needed a preload of 27. ± 0.6 mN (*n* = 8) vs. 30. ± 0.4 mN (*n* = 5, *p* < 0.01) for segments of old mice. Probably, this is due to the higher wall thickness of aortas isolated from aged mice.

The advantage of the ROTSAC setup is related to (i) the ability to independently control mechanical and pharmacological stimulation, (ii) the ability to expose the vessel to a physiological stretch rate, (iii) the ability to study several aortic segments of the same mouse, segments of different mice or different experimental protocols at the same time, and (iv) to compare effects of mechanical or pharmacological stimuli at the same extrapolated transmural pressure (isobaric measurements). These advantages might be especially relevant to aortic segments, given it is the significant visco-elastic behavior and (patho)physiological role in overall hemodynamics. Furthermore, the knowledge of the physiological role of VSMC contraction and relaxation in the aorta is still largely lacking, and data collected with the ROTSAC setup are expected to further contribute to a better understanding of the role of (contractile) cells in the aorta. It is evident that the ROTSAC setup cannot simply replace or be translated into the *in vivo* setting. Use of a biaxial pressurized myograph, which can be used for larger diameter vessels (300–1,000 μm) with (van der Bruggen et al., 2021) or without pulsation (Bersi et al., 2016), may result in additional experiments to demonstrate the (patho)physiological implications of the observations of the present study.

## Conclusion

The present study revealed that the ROTSAC equipment (Leloup et al., 2016) was suitable to study pressure dependency of arterial stiffness in different, well-controllable experimental conditions. Loading and unloading of aortic segments along a pressure range from 60 to 240 mm Hg and back, while continuously stretching the segments equivalent to a pulse pressure of 40 mm Hg to measure aortic compliance and stiffness, revealed that both the ECM and the VSMCs contribute to pressure-dependent stiffness hysteresis. Stiffness hysteresis phenomena were contraction, age, and ECM dependent, which suggests that VSMCs, in interaction with their ECM, contribute to a pressure-dependent pulse-dampening capacity of the aorta. The presumable role of focal adhesions as the link between VSMC cytoskeleton, integrins, and ECM in aortic stiffness hysteresis in the pulse-dampening capacity of the aorta might be a subject for future studies.

## Data Availability Statement

The raw data supporting the conclusions of this article will be made available by the authors, without undue reservation.

## Ethics Statement

The animal study was reviewed and approved by Ethical Committee of the University of Antwerp.

## Author Contributions

SD, AL, and PF contributed to the conception and design of the study performed the statistical analysis and wrote the first draft of the manuscript. All authors contributed to manuscript revisions, read and approved the submitted version.

## Funding

This work was supported by the University of Antwerp (GOA-BOF, grant 33931). AL was fellow of the FWO-Flanders (Belgium).

## Conflict of Interest

The authors declare that the research was conducted in the absence of any commercial or financial relationships that could be construed as a potential conflict of interest.

## Publisher's Note

All claims expressed in this article are solely those of the authors and do not necessarily represent those of their affiliated organizations, or those of the publisher, the editors and the reviewers. Any product that may be evaluated in this article, or claim that may be made by its manufacturer, is not guaranteed or endorsed by the publisher.

## Abbreviations

VSMC: vascular smooth muscle cell
ECM: extracellular matrix
ROTSAC: Rodent Oscillatory Set-up for Arterial Compliance
W: width of the aortic segment (as mounted in the ROTSAC set-p)
D: calculated diameter
L: length of the aortic segment
E_p_: Peterson elastic modulus
PP: pulse pressure
PE: phenylephrine
L-NAME: 300-μM NΩ-nitro-l-arginine methyl ester
RM ANOVA: repeated measures ANOVA.
