# Supplementary material for: Aortic Stiffness Hysteresis in Isolated Mouse Aortic Segments Is Intensified by Contractile Stimuli, Attenuated by Age, and Reversed by Elastin Degradation
Source: Front Physiol. 2021 Sep 28;12:723972. doi: 10.3389/fphys.2021.723972 (PMC8507434; doi:10.3389/fphys.2021.723972)
Supplement: Supplementary file 1 [file Data_Sheet_1.DOCX]

**Supplementary material:**

Supplementary figure 1:


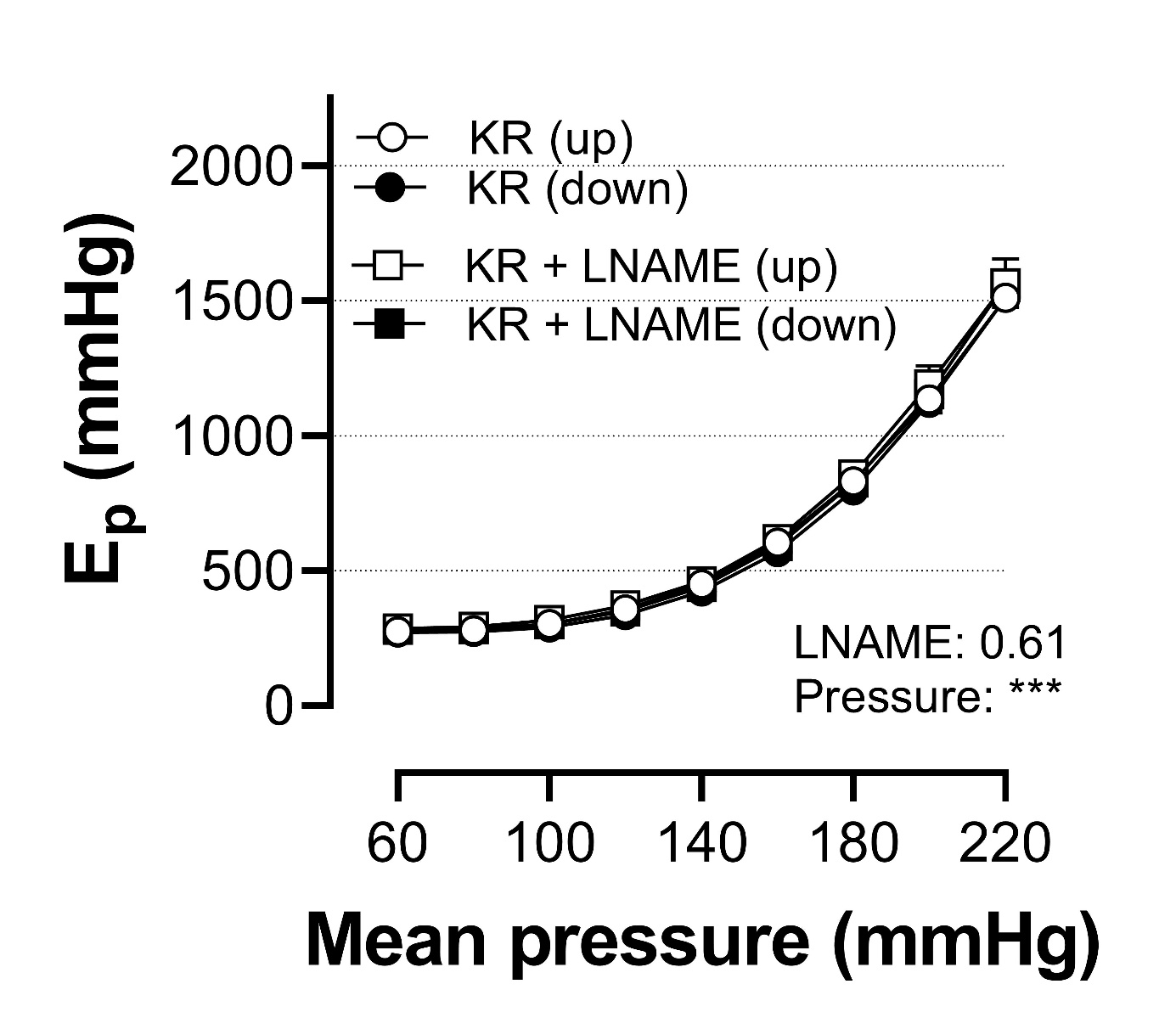


**Supplementary figure 1:** **Effect of L-NAME on the E_p_-pressure curves.** Segments of 8 mice were treated without (circles) or with (squares) 300 µM L-NAME. Starting at diastolic and systolic preload, according to 80 and 120 mm Hg, the preloads were adapted to obtain diastolic and systolic pressure of 40 and 80 mm Hg and subsequently increased by 20 mm Hg up to 200-240 mm Hg or higher (loading, up). Thereafter, the pressure was decreased back to 40-80 mm Hg (unloading, down) and finally to 80-120 mm Hg. At each mean pressure range E_p_ was measured and plotted against mean pressure. ). Two way RM ANOVA with Sidak’s multiple comparison test (LNAME versus KR) ***: p<0.001

Supplementary figure 2:


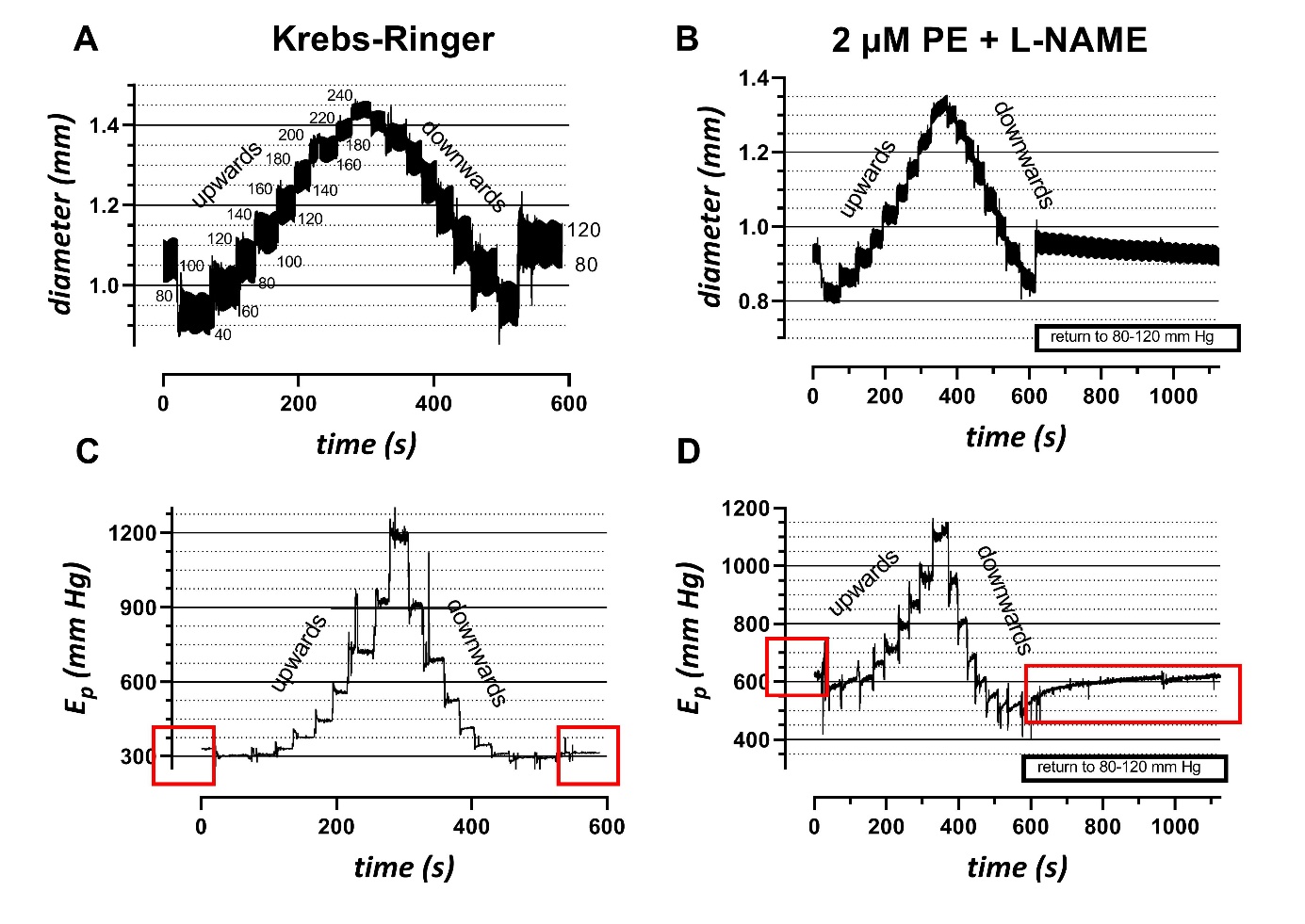


**Supplementary figure 2: Reversibility of hysteresis phenomena. Example of a pressure protocol in baseline conditions (KR, A, C) and following contraction with 2 µM PE in the presence of 300 µM L-NAME (B, D). Different pressure steps were applied on top of the cyclic stretch according to 40 mm Hg. Diastolic and systolic diameters (A, B) and E_p_ values (C, D) were determined for each pressure step. In the presence of PE and L-NAME, diameters and E_p_ were further followed and determined after the last step to the “control 80-120 mm Hg (indicated as return to 80-120 mm Hg) and data shows the slow (time constants of about 5 minutes) return of diameters and E_p_ to values comparable with values before the pressure steps.**
